# Responses of the tree peony (*Paeonia suffruticosa*, Paeoniaceae) cultivar ‘Yu Hong’ to heat stress revealed by iTRAQ-based quantitative proteomics

**DOI:** 10.1186/s12953-022-00202-5

**Published:** 2022-12-29

**Authors:** Jin Ma, Qun Wang, Ling-Ling Wei, Yu Zhao, Guo-Zhe Zhang, Jie Wang, Cui-Hua Gu

**Affiliations:** 1 College of Landscape and Architecture, Zhejiang Agriculture & Forestry University, Hangzhou, 311300 China; 2 Zhejiang Provincial Key Laboratory of Germplasm Innovation and Utilization for Garden Plants, Zhejiang Agriculture & Forestry University, Hangzhou, 311300 China; 3 Key Laboratory of National Forestry and Grassland Administration On Germplasm Innovation and Utilization for Southern Garden Plants, Zhejiang Agriculture & Forestry University, Hangzhou, 311300 China; 4 Institute of Ecological Civilization, Zhejiang Agriculture & Forestry University, Hangzhou, 311300 China; 5 School of Humanities & Social Sciences, Beijing Forestry University, Beijing, 100083 China; 6 Kunpeng Institute of Modern Agriculture at Foshan, Guangdong Foshan, 528200 China; 7 College of Science, Health, Engineering and Education, Murdoch University, Perth, WA 6150 Australia

**Keywords:** Ornamental plant, Abiotic stress, Physiological response, Omics analyses, Heat-responsive proteins

## Abstract

Horticulture productivity has been increasingly restricted by heat stress from growing global warming, making it far below the optimum production capacity. As a popular ornamental cultivar of tree peony, *Paeonia suffruticosa* ‘Yu Hong’ has also been suffering from heat stress not suitable for its optimal growth. To better understand the response mechanisms against heat stress of tree peony, investigations of phenotypic changes, physiological responses, and quantitative proteomics were conducted. Phenotypic and physiological changes indicated that 24 h of exposure to heat stress (40 °C) was the critical duration of heat stress in tree peony. The proteomic analyses revealed a total of 100 heat-responsive proteins (HRPs). According to bioinformatic analysis of HRPs, the heat tolerance of tree peony might be related to signal transduction, synthesis/degradation, heat kinetic proteins, antioxidants, photosynthesis, energy conversion, and metabolism. Our research will provide some new insights into the molecular mechanism under the response against the heat stress of tree peony, which will benefit the future breeding of heat-resistant ornamental plants.

**Supplementary Information:**

The online version contains supplementary material available at 10.1186/s12953-022-00202-5.

## Introduction

Global temperatures have been rising in recent decades mainly due to the rapid accumulation of greenhouse gas in the atmosphere [1], making the higher temperature key to plant growth and development as an essentially abiotic limited factor [2, 3]. Tree peony (*Paeonia suffruticosa*, Paeoniaceae) is a deciduous shrub widely distributed around the world [4, 5]. As an excellent ornamental plant, tree peony has been adored by people for its elegant appearances and gorgeous colors, and even awarded the ‘king of flowers’ in China [6–9]. In cool climates, tree peony can grow vigorously under trees and along riverbanks. However, temperatures over 26 °C in the summer will interrupt its normal growth, which severely limits their use as ornamental plants. Therefore, improving new tree peony cultivars with heat-resistance is of great importance for ornamental breeding.

How serious the plant is affected under certain stress can be accurately reflected by some physiological and biochemical indices. Previous research has shown that plants respond to external stress by enhancing antioxidant enzyme activities, like peroxidase and superoxide dismutase, to remove reactive oxygen species (ROS) from the body and weaken lipid oxidation in the cell membrane [10, 11], as well as the synthesizing osmoregulatory substances like proline and soluble protein to maintain the water balance inside and outside the cell [12, 13]. Furthermore, the level of malondialdehyde and relative conductivity can be used to characterize the severity of cell damage [14, 15]. However, most research regarding tree peony heat tolerance focus on physiological and biochemical changes, while few are on molecular biology. A lack of understanding of the molecular mechanism of tree peony heat tolerance makes it necessary to perform molecular investigations on its heat stress response.

Proteins are critical components of cellular responses, and changes of proteomic facing stress can reveal how plants respond to stress [16, 17]. Proteomics has become a cornerstone of systematic biology and are effective in addressing numerous biological problems in recent decades along with the development of protein sequencing technology [1, 18]. Isobaric tags for relative and absolute quantification (iTRAQ) together with tandem mass spectrometry (MS/MS) has been developed as a superior choice for precise quantification of diverse proteins, especially the lowly expressed proteins [19–21]. For example, iTRAQ combined with MS/MS was used to investigate differentially expressed proteins associated with shoot dormancy release in tree peony under low-temperature treatment [6]; similarly iTRAQ combined with MS/MS was used to investigate the molecular mechanism of hybridization incompatibility in tree peony and its relatives [22]. However, to our knowledge, there have been no reports on the use of proteomic approaches to evaluate heat tolerance in tree peony.

Leaves are the principal trophic organs that directly sense temperature changes in the environment and have phenotypic plastic responses to such changes [23]. Temperature fluctuations also affect leaf photosynthesis and transpiration, which are the foundations of plant growth and development [24]. As a result, research focusing on the impacts of high temperatures on leaves will benefit the understanding how plants respond to heat stress. Herein, the iTRAQ-MS/MS and corresponding biological analyses to characterize the proteomic changes of the heat-tolerant tree peony ‘Yu Hong’ leaves under heat stress, and to reveal the responsive mechanism of tree peony under heat stress at the protein level, and to provide a theoretical basis for the future cultivation of new heat-tolerant tree peony germplasm.

## Material and methods

### Plant materials

Tree peony seeds were collected from the National Color Family Farm in Jinhua, Zhejiang Province, and the annual live seedlings of Jiangnan tree peony cultivar ‘Yu Hong’ with high heat tolerance were used in this study.

The seeds were dried naturally indoors after sampling, soaked in water for 24 h, plump seeds were chosen to be sterilized with 0.5 percent potassium permanganate solution, and sowed in the Zhejiang A & F University base in September 2018. Selected seedlings with steady growth patterns were transferred into pots with a diameter of 50 cm in March 2019. Each pot contained one plant. Routine management was conducted during the slow seedling stage.

### Seedling stress treatment

Live seedlings with good uniformity and growth were chosen and separated into two groups: control (ambient treatment at 25 °C) and experimental (high temperature treatment at 40 °C). Four treatment gradients (0, 12, 24, and 36 h) were set in the control group and the experimental group respectively, and three biological replicates were set in each gradient, a total of 24 pots of plant materials. After 0, 12, 24, and 36 h of treatment, the leaf tissues were collected, marked, and rapidly placed in liquid nitrogen for subsequent measurement of the physiological indicators and protein extraction. During the experiment, the air humidity in the incubator was set to 80%, the light intensity was set to 4000 LX, and the light time was set to 12 h of light and 12 h of darkness.

### Measurement of physiological parameters in seedlings

The acidic ninhydrin colorimetric technique was used to determine the content of proline (Pro) [25, 26]. The guaiacol method was used to determine the content of peroxidase (POD) [27]. The thiobarbituric acid (TBA) method was used to determine the content of malondialdehyde (MDA) [28]. The nitrogen blue tetrazolium (NBT) photochemical reduction method was used to measure the content of superoxide dismutase (SOD) [29]. The Coomassie bright blue technique was used to determine the content of soluble protein SP [30]. The relative electric conductivity (REC) of leaves after various treatments can be measured using a conductivity meter (DDS-307A, Shanghai Yoke Instrument Co., Ltd.). REC is equal to (S1/S2) 100 after measuring the initial electrolyte leakage (S1), heating the sample to 100℃, and measuring the final electrolyte leakage (S2) at room temperature.

### Protein extraction, digestion, iTRAQ labeling, and data analysis

Protein extraction was performed according to the trichloro-acetic acid/acetone precipitation method [31] for the three materials under each treatment, without mixing. Samples were ground into powder in liquid nitrogen and dissolved in lysis buffer (pH = 8) containing 8 mol/L urea, 0.2% SDS, and 50 mmol/L Tris–HCl, followed by 5 min of ultrasonication on ice. The lysate was centrifuged at 13 000 r/min for 20 min at 4 °C and the supernatant was transferred to a clean tube. Extracted proteins were reduced with 2 mmol/L DTT for 1 h at 55 °C and subsequently alkylated with sufficient iodoacetic acid for 1 h at 25 °C. Then samples were mixed with 5 volumes of precooled acetone by vortexing and incubated at –20 °C for 3 h. Samples were then centrifuged and the precipitation was collected. After washing twice with cold acetone, the pellet was dissolved by dissolution buffer (pH = 8.5) containing 8 mol/L urea and 0.1 mol/L TEAB. Total protein concentration was determined by the Bradford protein assay [32].

Enzymatic digestion of trypsin to sample protein at a 20:1 ratio. After 15 h of digestion at 37 °C, peptides were desalted with a C18 cartridge to remove high-concentration urea. The six labeling reagents iTRAQ115, iTRAQ116, iTRAQ117, iTRAQ118, iTRAQ119, and iTRAQ121 of the iTRAQ reagent-8plex complex kit (Sigma, USA) were used to label the experimental (TE1, TE2, TE3) and control (CK1, CK2, CK3) desalting peptides, respectively, according to the manufacturer's instructions. The labeling reagent and peptides were dissolved with 70 µL of isopropanol and 20 µL of 0.5 mol/L TEAB, respectively. One unit of labeling reagent was used for 100 μg of peptides. After incubation for 1 h at room temperature, the reaction was stopped with 100 µL of 50 mmol/L Tris–HCl (pH = 8). Individually iTRAQ-labeled peptides were mixed in equal volumes, and then desalted and lyophilized. iTRAQ-labeled peptide mix was fractionated using a XB ridge Peptide BEH C18 column (25 cm × 4.6 mm, 5 µm) on a L3000 HPLC Cperating System at a rate of 1 mL/min; the column oven was set as 50 °C. Buffer A (2% acetonitrile, pH = 10) and B (98% acetonitrile, pH = 10) were used to develop a gradient for elution. The solvent gradient was set as follows: 0–3 min, 100% A, 0% B; 3–21 min, 95% A, 5% B; 21–27 min, 80% A, 20% B; 27–33 min, 70% A, 30% B; 33–58 min, 0% A, 100% B. Based on the peak pattern, 12 fractions were chosen. All fractions were lyophilized and reconstituted in 0.1% formic acid. Proteomics analyses were processed using an EASY-nLCTM 1200 UHPLC system (ThermoFisher) coupled with a Q Exactive HF-X mass spectrometer (ThermoFisher). A total of 2 µg of total peptides reconstituted in 0.1% formic acid was injected onto an Acclaim PepMap100 C18 NanoTrap column (100 µm × 2 cm, 5 µm). Then peptides were separated on a Reprosil-Pur 120 C18-AQ analytical column (150 µm × 15 cm, 1.9 µm), at a flow rate of 600 nL/min with a 5–100% linear gradient of eluent B (0.1% formic acid in 80% acetonitrile) in eluent A (0.1% formic acid in H2O). The detailed solvent gradient listed as follows: 10% B, 90% A, 2 min; 30% B, 70% A, 49 min; 50% B, 50% A, 2 min; 90% B, 10% A, 2 min; 100% B, 5 min.

Q Exactive HF-X mass spectrometer was operated in positive polarity mode with a capillary temperature of 320 °C and spray voltage of 2.3 kV. Full MS scans ranging from 350 to 1500 m/z were acquired at a resolution of 60 000 (at 200 m/z) with a maximum ion injection time of 20 ms and an automatic gain control target value of 3 × 106. The 40 most abundant precursor ions from the full MS scan were selected for fragmentation using higher energy collisional dissociation fragment analysis at a resolution of 15,000 (at 200 m/z) with a normalized collision energy of 32%, an automatic gain control target value of 1 × 105, an intensity threshold of 8.3 × 103, the dynamic exclusion parameter of 60 s and a maximum ion injection time of 45 ms.

Using the search engines, the obtained spectra from each fraction were individually compared to the Uniprot Oryza sativa Database from uniprotOryzasativa. 2.1 of Proteome Discoverer (PD 2.1, ThermoFisher, USA). On the peptide and protein levels, a protein was recognized as a protein having at least one distinct peptide (FDR ≤ 0.01), respectively. Different protein groups were created from proteins with similar peptide compositions that could not be distinguished based on an MS/MS study. During the search, iTRAQ quantification was done using iTRAQ 8-plex. The protein quantitation results were statistically analyzed by *t*-test, and the significant ratios, defined as *P* < 0.05 and fold change (FC) ≥ 1.5 or ≤ 0.67, were considered heat-responsive proteins (HRPs).

### Bioinformatics analysis

The sequences of HRPs were compared with three public databases: KEGG (Kyoto Encyclopedia of Genes and Genomes, http://www.genome.jp/kegg), COG (Clusters of Orthologous Groups of proteins, http://www.ncbi.Nlm.nih.gov/COG), GO (Gene Ontology, http://geneontology.org/). The Protein Blast (https://blast.ncbi.nlm.nih.gov/Blast.cgi) was used for sequence similarity comparison.

### Statistical analysis

One-way analysis of variance (ANOVA) with Duncan’s multiple range tests was conducted on the data, and *p* < 0.05 was considered significant. *t*-test analysis on protein quantitative results, *p* < 0.05 was considered significant. All the above analyses were accomplished with the IBM SPSS Statistics 21.0 software package (IBM Corporation, Armonk, NY, USA). All figures were drawn with the Origin 21.0 software (OlriginLab Co., Northampton, MA, USA).

## Results

### Phenotype and physiological responses to heat stress

To investigate the critical time of heat stress treatment for ‘Yu Hong’, phenotypic and physiological indices were recorded at 0, 12, 24, and 36 h of after heat treatment, respectively.

**Fig. 1:**
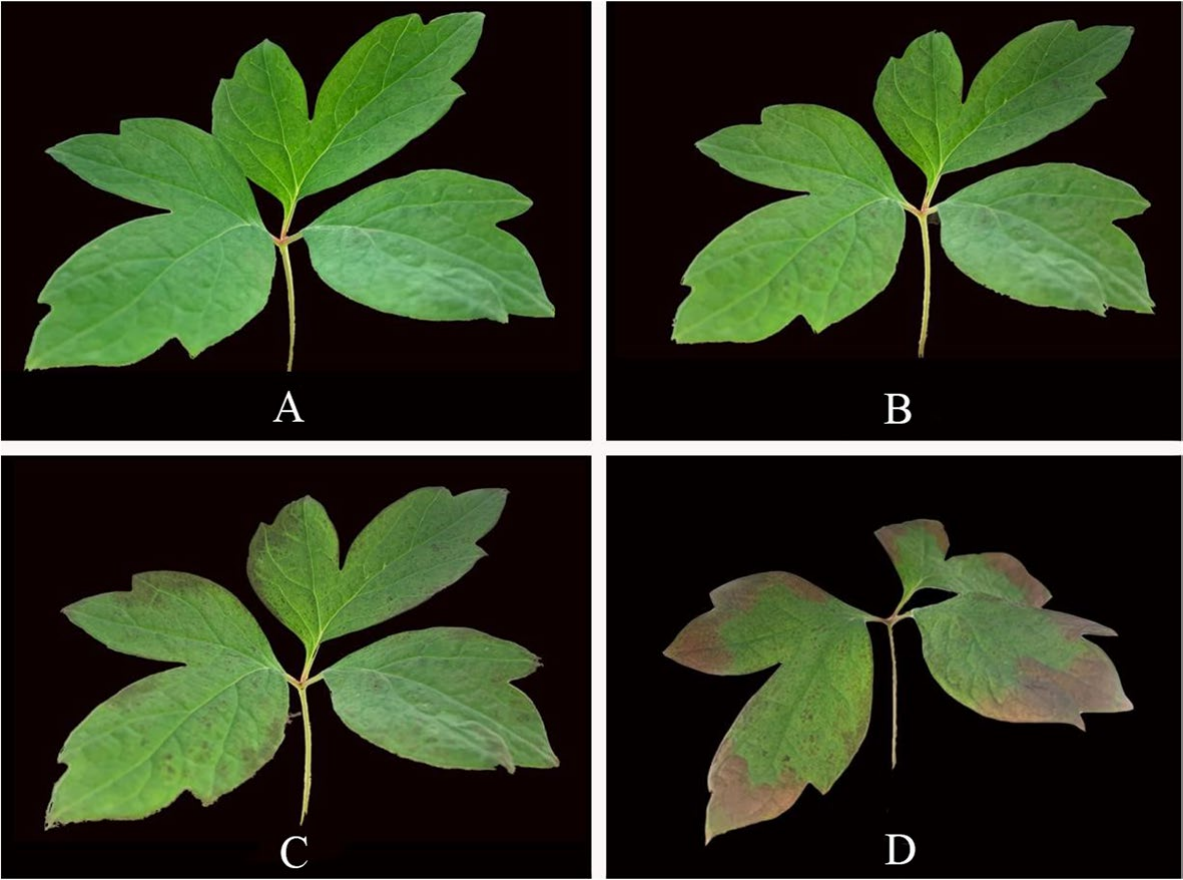
Phenotypic changes of ‘Yu Hong’ leaves under heat stress for 0, 12, 24, and 36 h, respective, indicated by A to D

The morphology of ‘Yu Hong’ leaves changed significantly as the high temperature treatment duration was extended. Compared to treatment 0 h (Fig. 1A), slight brown spots appeared on the edge of the leaves after 12 h of heat stress (Fig. 1B), The brown spots on the edge of leaves increased significantly after 24 h of heat stress compared to 12 h, and the stems and leaves were slightly yellowed (Fig. 1C). The seedlings developed much worse and the leaves showed obvious heat damage symptoms after 36 h of heat stress, with browning of the leaf edges, curling and crinkling of the leaves, narrowing of the leaf area, and plants on the verge of withering (Fig. 1D).

**Fig. 2:**
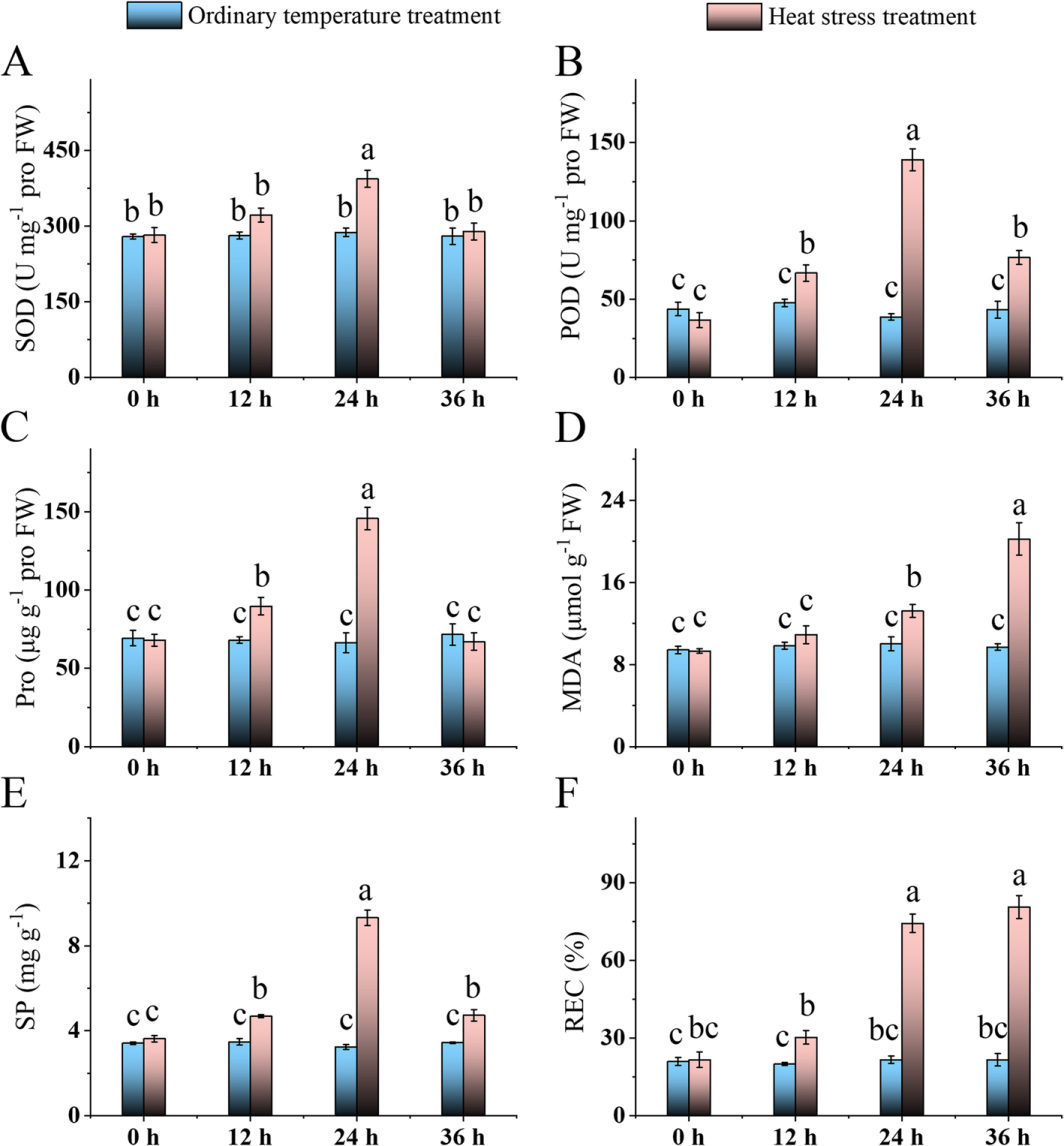
Changes of physiological parameters of ‘Yu Hong’ leaves with treatment time. The physiological parameters include superoxide dismutase content (**A**), peroxidase content (**B**), proline content (**C**), malondialdehyde content (**D**), soluble protein content (**E**), and the relative electric conductivity (**F**). Different letters indicate a significant difference (*p* < 0.05) using Duncan’s multiple range test

The SOD activity, POD activity, proline content, and SP of ‘Yu Hong’ leaves showed a trend of increasing and then decreasing with the extension of treatment time, MDA content was gradually increasing, and the REC showed a trend of increasing and then stabilizing under heat stress. Among them, SOD activity increased significantly after 24 h of heat stress, then recovered to the treatment 0 level after 36 h. After 12 h of high temperature treatment, POD activity and SP content began to rise dramatically, peaked after 24 h, and then recovered to pre-treated levels after 36 h. After 12 h of high temperature treatment, proline content grew considerably, peaked after 24 h, and then recovered to pre-treatment levels after 36 h. After 24 h of high temperature treatment, the MDA level began to rise rapidly, peaking at 36 h. After 24 h of treatment, the REC peaked and remained steady as the treatment time was extended (Fig. 2).

### Responses of HRPs to heat stress

According to the above changes of leaf phenotype and leaf physiological parameters of ‘Yu Hong’, 24 h of heat stress was the critical duration of ‘Yu Hong’ in response to heat stress. So, we chose the material after 24 h of high temperature treatment for further iTRAQ studies.

**Fig. 3:**
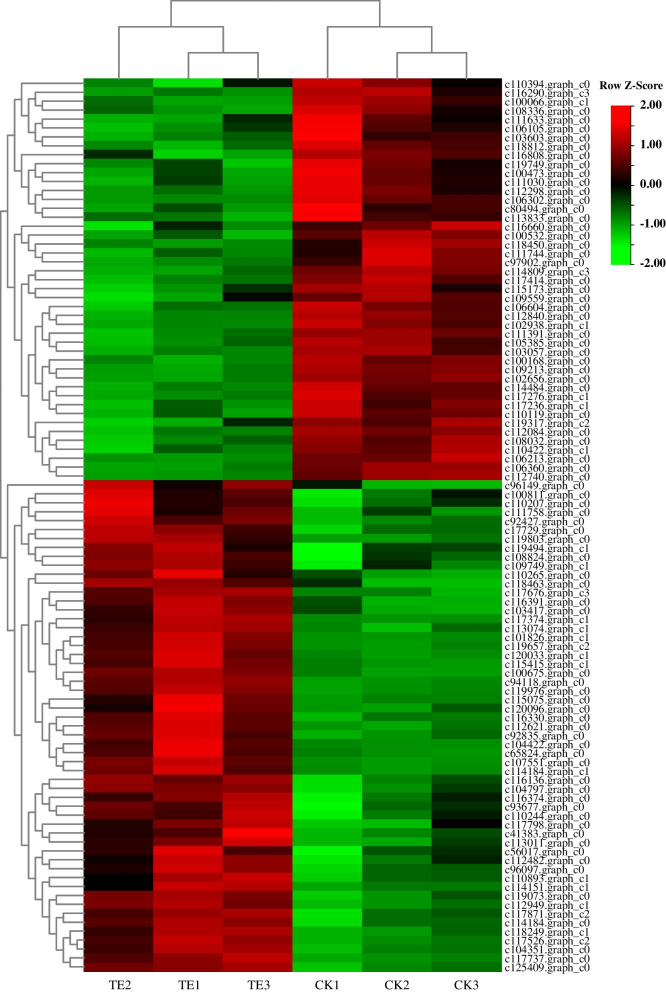
The change of HRPs in response to heat stress based on the proteomics data. CK1, CK2, CK3 represent three control groups, and TE1, TE2, TE3 represent three experimental groups

iTRAQ screening revealed a total of 1916 proteins. Compared to normothermic control samples, a total of 100 HRPs were identified under heat stress (Fig. 3).

### Functional analysis of HRPs in response to heat stress

**Fig. 4:**
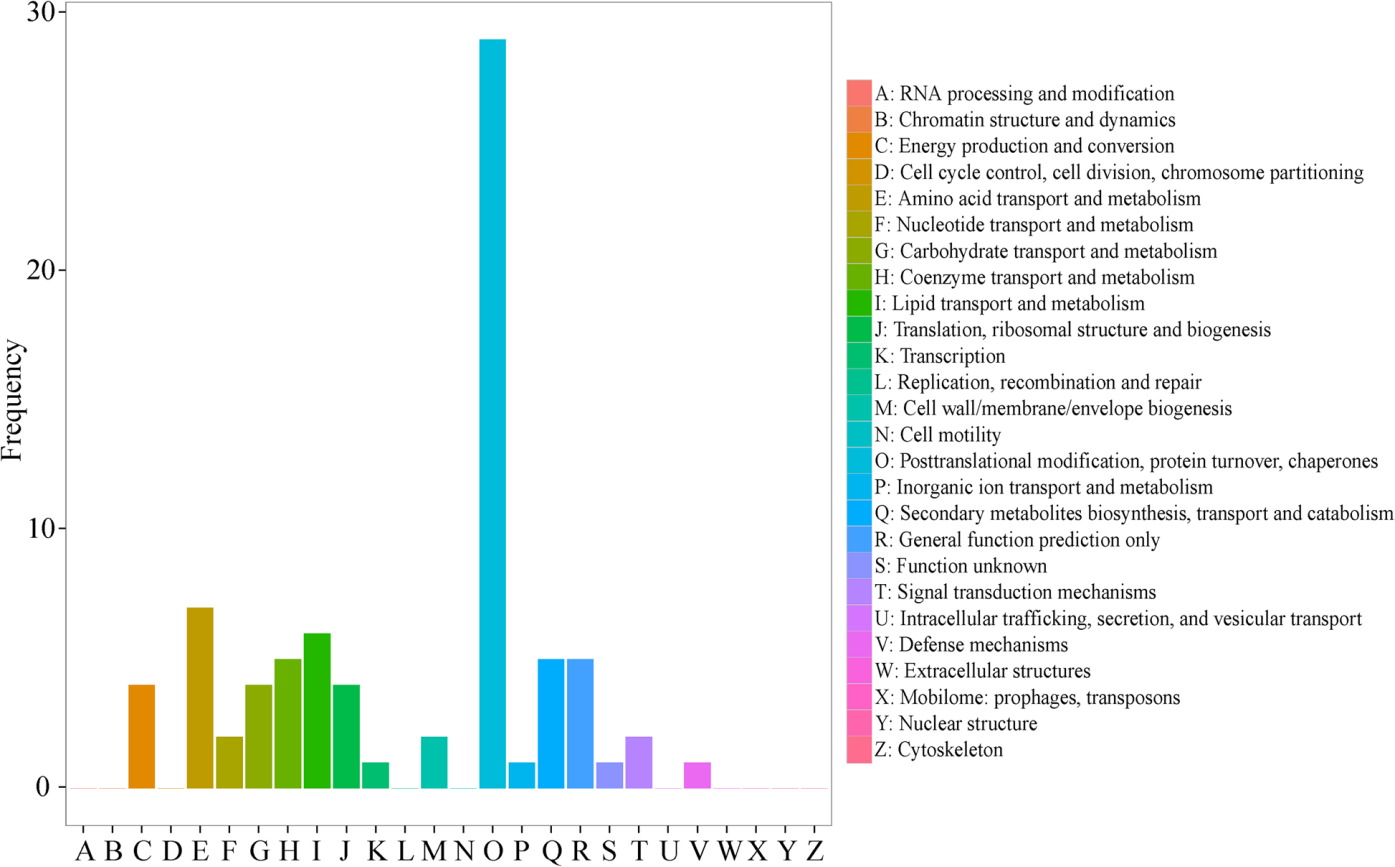
COG annotation analysis of HRPs. Frequency indicates the number of HRPs in each COG functional classification

Of the 100 HRPs, 79 HRPs were categorized into 16 categories using COGs database. The largest group was Posttranslational modification, protein turnover, chaperones, followed by Amino acid transport and metabolism, Lipid transport and metabolism, Coenzyme transport and metabolism, Secondary metabolites biosynthesis, transport and catabolism, General function prediction only, Translation, ribosomal structure and biogenesis, Energy production and conversion and Carbohydrate transport and metabolism (Fig. 4).

**Fig. 5:**
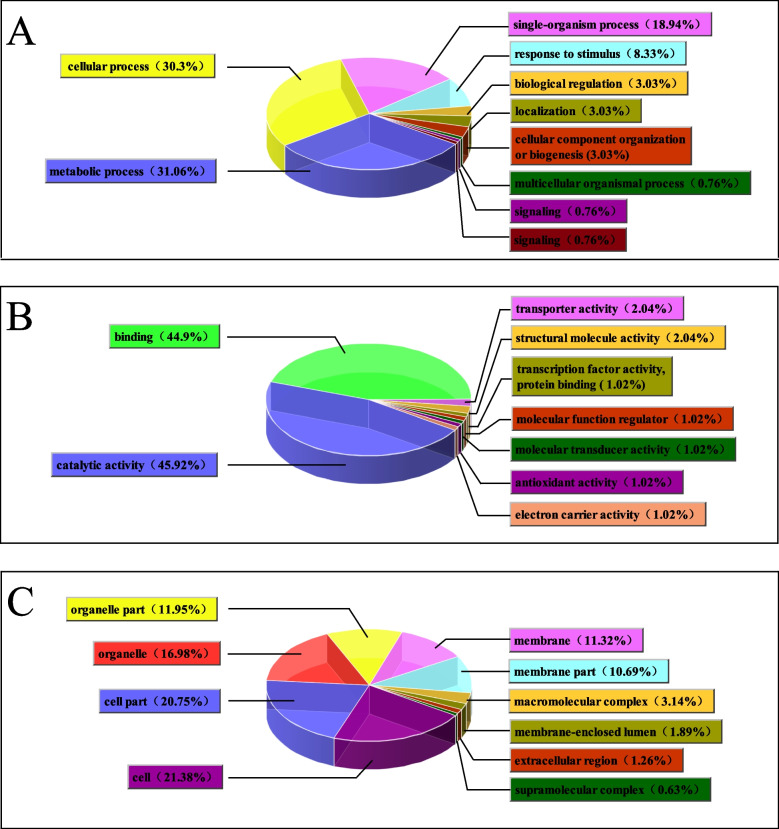
GO annotation of HRPs. Biological process (**A**), Cellular component (**B**), Molecular function (**C**)

To further characterize all heat-responsive proteins, these HRPs identified were subjected to GO analysis. Of the 100 HRPs identified under heat stress, 74 HRPs were annotated into three groups as cellular component (159), molecular function (98), and biological process (132). GO analysis showed that cell (34), cell part (33), and organelle (27) were the major cellular component terms; catalytic activity (45) and binding (44) were the dominant molecular function terms; and metabolic process (41), cellular process (40), and single-organism process (25) were the most dominant biological process terms (Fig. 5).

**Fig. 6:**
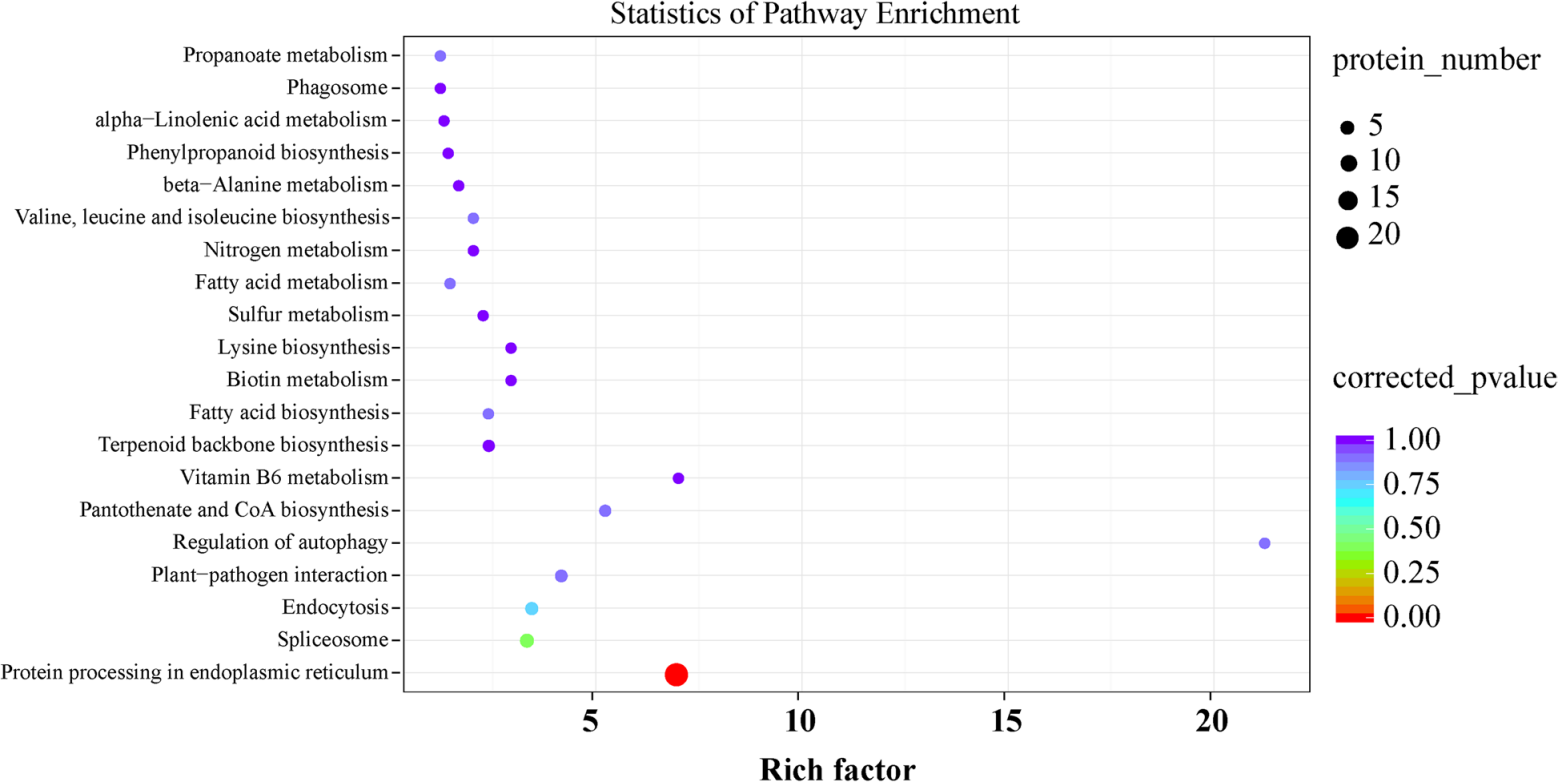
KEGG pathway enrichment of HRPs

To further investigate the major metabolic pathways responding to heat stress, KEGG enrichment analysis was carried out with HRPs. KEGG pathway enrichment showed that 42 HRPs were annotated into 33 pathways. The protein processing pathway in endoplasmic reticulum is the most, with 22 HRPs (Fig. 6).

**Fig. 7:**
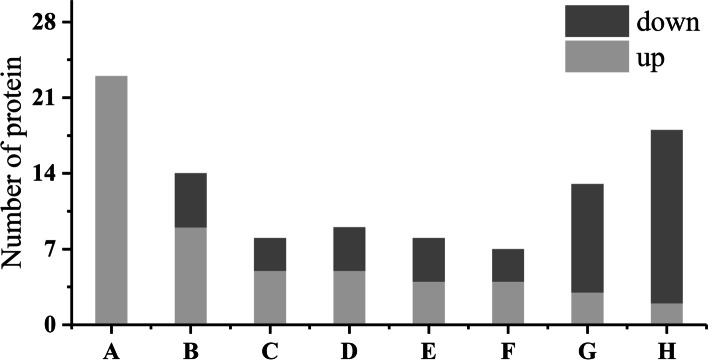
Classification of HRPs into eight categories. **A**, Heat shock protein; **B**, Protein synthesis/degradation; **C**, Antioxidants; **D**, Energy conversion; **E**, Signal transduction; **F**, Photosynthesis; **G**, Metabolism; and **H**, Other unknown functional HRPs

Through the similarity comparison of the amino acid sequences of heat response proteins through the Protein Blast tool of the NCBI online website, the 100 HRPs were classified into eight categories, i.e., a) protein synthesis/degradation (14), b) heat shock protein (23), c) defense and antioxidants (8), d) energy production and conversion (9), e) signal transduction (8), f) photosynthesis (7), g) metabolism (13), and h) other unknown functional HRPs (18) (Fig. 7). Details of HRPS can be found in Addendum 1.

All 23 HRPs classified as heat shock proteins were up-regulated. Among the 14 HRPs involved in protein synthesis, processing and degradation, 9 were up-regulated and 5 were down-regulated. There were 5 up-regulated and 3 down-regulated HRPs in those involved in antioxidants. Among the 9 HRPs involved in energy conversion, 5 were up-regulated and 4 were down-regulated. Among 7 HRPs involved signal transduction, 4 were up-regulated and 3 were down-regulated. In metabolism there were 3 up-regulated and 10 down-regulated HRPs. As to the 18 HRPs with unknown functions, 2 were up-regulated, and 16 were down-regulated (Fig. 7).

### Heat stress response model

**Fig. 8:**
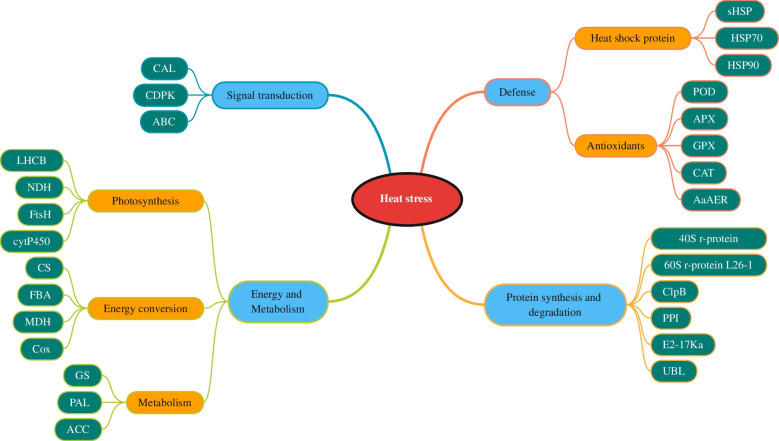
The model of tree peony response to heat stress. Cal, Calmodulin; CDPK, Calcium-dependent protein kinase; ABC, ABC transporter family member; LHCB, Chlorophyll a / b binding protein; NDH, NADH dehydrogenase; FtsH, FtsH protease; cytP450, Cytochrome P450; CS, Citrate synthase; FBA, Fructose- 1,6- bisphosphate aldolase; MDH, Malate dehydrogenase; Cox, Cytochrome C oxidase; GS, Glutamine Synthetase; PAL, Phenylalanine ammonia lyase; ACC, Acetyl-CoA catablerboxylase; sHSP, Small heat shook protein; HSP70, Hsp70 family protein; HSP90, Hsp90 family protein; POD, Peroxidase; APX, Ascorbate peroxidase; GPX, Glutathione peroxidase; CAT, Catalase; AaAER, 2-alkenal reductase; 40S r-protein, 40S Ribosomal protein; 60S r-protein, 60 s ribosomal protein L26-1; ClpB, Chaperone-protein ClpB; PPI, Peptidylprolyl isomerase; E2-17Ka, Ubiquitin-binding enzyme E2-17Ka; UBL, Ubiquitin-like family protein

Based on the results of proteomic analyses, we comprehensively identified the key HRPs of tree peony and proposed a heat response model of proteins in tree peony (Fig. 8). HRPs were divided into three levels in this model. The heat response protein functions were divided into four groups on the first level (blue) including signal transduction, energy conversion and metabolism, protein synthesis/degradation, and defense. The second level (Orange) was a further division of energy conversion and metabolism, and defense, among which the heat stress proteins and antioxidants were under defense while photosynthesis, energy conversion, and metabolism under energy and metabolism. The heat response proteins that corresponded to each functional pathway were the third level (green) in the model.

## Discussion

Heat stress will cause an imbalance between ROS generation and antioxidant defenses, resulting in an enrichment of ROS in plants, which will damage plant cells and even inhibit plant growth and secondary metabolite induction [33, 34]. MDA is a lipid peroxidation product that is considerably elevated if the lipids in plant cells are damaged [35]. Pro, SP, POD, SOD, and REC are also crucial indicators of cell oxidative damage, with higher values indicating that the plant is under more severe stress [21]. Here, compared to 12 h of heat stress, tree peony leaves were on the verge of wilting at 24 h of heat stress (Fig. 1), while leaves were already browning and wilting at 36 h of heat stress (Figs. 2A, B). More importantly, all physiological indicators except malondialdehyde reached their highest levels after 24 h of heat stress, and the malondialdehyde content started to increase significantly after 24 h of heat stress. This shows that 24 h is the optimum duration of treatment for tree peony ‘Yu Hong’. The results of the proteomic analysis of the heat response under 24 h of heat stress will be discussed further below.

### HRPs involved in signal transduction

Stress perception, signal transduction, and the expression of certain stress-related genes and metabolites all play a role in plant adaptation to environmental stress [36]. Signal transduction is a complex regulatory system in plants that plays an important role in sustaining their normal growth and development in response to abiotic stresses [37, 38]. The importance of Ca^2+^ as a second messenger in the signal transduction pathway is well understood [39]. Abiotic stress causes a transitory rise in cytoplasmic Ca^2+^, either through exosomal inward flow or the release of internal reserves [40]. Calmodulin is the most well-known Ca^2+^ sensor in signal transduction, and it is involved in heat shock signaling [37]. Calcium-dependent protein kinase is a serine/threonine protein kinase with c-terminal calmodulin-like structural domain at its c-terminus, making it both a Ca^2+^ sensor and an effector of Ca^2+^ signal transduction [39, 40]. The fact that these two proteins (c109749.graph_c1, c119803.graph_c0) are up-regulated in our study shows that they are involved in signal transduction mechanisms in response to heat stress.

Ca^2+^ signaling is involved in the transport of membrane proteins and vesicles in plant cells, in addition to directing ion transport [41]. Vesicular transport is well established as a key mode of communication between organelles in cells. Most of the vesicular transport is mediated by soluble N-ethylmaleimide-sensitive factor attachment protein receptors (SNAREs), and highlight fusion protein is a subfamily of SNAREs proteins [42–44]. ABC transporter family member is also a widely distributed transporter protein in plants, and ABC functions have been shown to be involved in signal transduction for physiological processes such as hormone transport, pathogen resistance, abiotic stressors, and detoxifying [45, 46]. The fact that these two proteins (c116136.graph_c0, c113074.graph_c1) up-regulated in our study shows that they were also involved in signal transduction mechanisms in response to heat stress.

### HRPs involved in protein synthesis/degradation

Protein synthesis is inhibited by heat stress [17]. Plant responses to environmental stress rely heavily on maintaining a balance between intracellular protein production and breakdown [47]. A variety of proteins involved in protein synthesis were shown to be down-regulated in this study, including 1 aspartate protease family protein (c117236.graph_c1), 1 trigger factor protein (c108032.graph_c0), 1 peptidine family protein (c117276.graph_c1), 1 subtilisin-like serine protease (c92427.graph_c0), and 1 protein disulfide isomerase (c112740.graph_c0). Interestingly, many proteins were also up-regulated. Ribosomes is a huge macromolecular ribosomal protein that catalyze protein synthesis in cells and play a vital role in cell growth, development, and differentiation [21]. The effectiveness and stability of the ribosome are directly influenced by the expression of ribosomal proteins [48]. In this study, we identified 1 40S ribosomal protein (c116290.graph_c3) and 1 60 s ribosomal protein L26-1 (c110207.graph_c0) up-regulation. We also discovered the up-regulated expression of 2 chaperone-protein ClpB (c103417.graph_c0, c118249.graph_c1) and 3 peptidylprolyl isomerase (c115415.graph_c1, c111758.graph_c0, c106105.graph_c0). The most immediate hazard of heat stress is the misfolding of proteins, which causes loss of function and consequently harm [47]. So, the elimination of misfolded proteins is very important for protease stability and prevention of protein lesions [49]. The elimination of misfolded proteins is essential for protease stabilization and prevention. PPI, a protein folding enzyme that catalyzes peptidyl proline cis–trans-isomerization, may also be involved in protein complex construction and disassembly, protein transport, and protein activity control [2, 47]. ClpB is a chaperone protein found in bacteria, fungi and plants that cooperate with other chaperone proteins to rescue misfolded proteins that have aggregated—an activity that helps cells survive heat shock and other stresses [50]. Furthermore, we discovered that the expression of 1 Ubiquitin-binding enzyme E2-17Ka (c104797.graph_c0) and 1 Ubiquitin-like family protein (c93677.graph_c0) involved in the ubiquitination process had increased. The ubiquitination process is a crucial step in the selective degradation of intracellular proteins [49, 51, 52]. Increased expression of these proteins helps maintain the balance between protein production and degradation while also enhancing thermotolerance.

### HRPs involved in stress defense

#### Heat shock protein

Plants need molecular chaperones to prevent the creation of misfolded proteins and maintain intracellular equilibrium in stressful situations [53]. HSPs are a type of molecular chaperone protein that helps plants protect themselves from stress and adapt to environmental changes [21, 54]. sHSP, HSP70 and HSP90 were all subsets of HSPs [55, 56]. Many studies have found that sHSP, HSP70, and HSP90 production was linked to plant heat tolerance, and that overexpression improves plant heat tolerance [57–60]. Our research found that all 23 HSPs discovered in the study, including 14 sHSP (c107551.graph_c0, c117526.graph_c2, c120033.graph_c1, c112621.graph_c0, c117374.graph_c1, c112949.graph_c1, c104422.graph_c0, c100675.graph_c0, c116391.graph_c0, c92835.graph_c0, c110265.graph_c0, c94118.graph_c0, c114184.graph_c1, c114184.graph_c0), 6 HSP70 (c119976.graph_c0, c65824.graph_c0, c110893.graph_c1, c17729.graph_c0, c112482.graph_c0, c114151.graph_c1), and 3 HSP90 (c101826.graph_c1, c119657.graph_c2, c120096.graph_c0), were upregulated. They clearly perform a crucial role as chaperone proteins in the tree peony's heat stress response.

#### Antioxidants

The equilibrium between the generation and scavenging of ROS is frequently disrupted by heat stress, which stimulates the formation of ROS, which can damage membrane systems and other cellular components [17]. Plants have evolved efficient enzymatic antioxidant systems to deal with the peroxidative damage of ROS during their long-term adaptive evolution [61]. POD (c100066.graph_c1), APX (c115075.graph_c0), GPX (c114484.graph_c0), and CAT (c116374.graph_c0), all of which are enzymatic antioxidants of the enzymatic antioxidant system, were among the up-regulated antioxidant enzymes in this study [23, 37, 38]. In response to heat stress, up-regulated production of these proteins is necessary for scavenging ROS.

Excess ROS can cause lipid oxidation, resulting in highly reactive lipid peroxidation-derived molecules like 4-hydroxy-2-nonenal, 4-hydroxy-2-hexenal, malondialdehyde, and acrolein, which are known as reactive carbonyl species (RCS) [62]. RCS in high quantities can cause irreversible cell damage and even death [63]. As a result, cellular harm might result from poor RCS clearance of reactive carbonyl molecules. We found 1 up-regulated 2-alkenal reductase (c118463.graph_c0), which detoxifies lipid-derived RCS by catalyzing the conversion of unsaturated bonds (C = C) to saturated bonds and contributes to plant cellular detoxification [64, 65].

### HRPs involved in energy and metabolism

#### Photosynthesis

Photosynthesis is the most basic physiological process for plant growth and development, as well as the most important source of energy for plant metabolism [38, 66, 67]. Photosynthesis is frequently regarded as the most thermosensitive physiological process due to its intricate molecular mechanism and the requirement for several enzymes to participate in its regulation [17, 21, 68]. When the external temperature exceeds the optimal adaption range of plants, photosynthesis diminishes, and this decline is linked to the suppression of the RuBisCO-activating enzyme [38]. Our findings support this conclusion since we discovered 2 down-regulated Rubisco. Interestingly, 1 chlorophyll a / b binding protein (c96149.graph_c0) [69], 1 NADH dehydrogenase (c116330.graph_c0) [70], 1 FtsH protease (c108824.graph_c0) [71], and 1 cytochrome P450 (c118450.graph_c0) [72] involved with the positive regulation of photosynthesis were also found and up-regulated in this study. We hypothesize that this is a long-evolved approach adopted by plants to cope with environmental stress, with the primary goal of maintaining photosynthetic efficiency and avoiding energy imbalances produced by stress.

#### Energy conversion

Respiration, the main metabolic pathway metabolism of carbohydrates, is an enzymatic oxidation reaction and is an important pathway for energy conversion in plants [37]. In this study, 1 citrate synthase (c104351.graph_c0), 1 fructose- 1,6- bisphosphate aldolase (c41383.graph_c0), 1 phosphoglycerate kinase (c117676.graph_c3) and 1 Malate dehydrogenase (c112840.graph_c0) were up-regulated, and both of them are important regulatory enzymes in glycolysis pathway and tricarboxylic acid cycle pathway in carbohydrate metabolism [73]. Promoting energy conversion is also an important measure to cope with heat stress. Furthermore, 1 up-regulated cytochrome C oxidase (c119073.graph_c0) was discovered in this study, which is found in the electron transport system of mitochondria and generates two molecules of water by transferring electrons to O^2−^. Because mitochondria, but not chloroplasts, create ROS, defects or shortages in this enzyme can be lethal to plants [74, 75]. This finding shows that shielding mitochondria from oxidative damage produced by heat stress can aid in heat stress management.

#### Metabolism

Heat stress, even for a short period of time, may alter plant metabolism, which in turn may affect plant responses to stress [76, 77]. Because nitrogen is a fundamental component of amino acid composition and serves a variety of other activities, it is an important factor in influencing plant development and stress resistance [78]. In this study, 1 glutamine Synthetase (c111030.graph_c0) and 1 phenylalanine ammonia lyase (c117414.graph_c0), both involved in nitrogen metabolism, were shown to be up-regulated [79]. This could be because under heat stress, inactive nitrogen metabolism can stifle amino acid synthesis, and a lack of specific amino acids can cause plant development to be thermally inhibited [78]. As a result, increasing nitrogen metabolism can aid in plant heat stress tolerance.

Fatty acids are typical ester compounds in plants that are classified as saturated or unsaturated depending on whether they include unsaturated bonds (C = C). Many studies have found that fatty acid desaturation is a key determinant of heat tolerance, possibly because unsaturated fatty acids may eliminate ROS and harmful chemicals while also improving cell membrane stability [80–82]. We discovered 1 up-regulated acetyl-CoA carboxylase (c117871.graph_c2) in our work, which is the initiator and key to regulate fatty acid biosynthesis through a series of condensation, dehydration and reduction reactions that can eventually desaturate or lengthen to produce a variety of different fatty acids for a variety of biological processes [83]. Thus, ACC helps to regulate fatty acid metabolism in response to heat stress.

## Conclusion

In this work, to uncover the molecular mechanisms underlying heat resistance in the tree peony cultivar ‘Yu Hong’ treated with normal and high temperature, physiological and proteomic changes were explored. A total of 100 differentially expressed proteins were identified as HRPs. These HRPs were found to be involved in signal transduction, protein synthesis/degradation, heat shock proteins, antioxidants, photosynthesis, energy conversion, and metabolism, according to bioinformatics study. Based on proteome changes, a model of tree peony response to heat stress was developed. In addition, other differentially expressed proteins with unknown functions were detected in this work, and their functions need to be confirmed in the future. Overall, our research provides valuable insights into the molecular mechanism of heat tolerance of tree peony, and may provide a useful reference for cultivating new varieties of heat resistant tree peony.

## Supplementary Information

**Additional file 1: Schedule 1.** Significantly expressed differential proteins under high-temperaturestress.

## Data Availability

The proteomics data used to support the findings of this study are available from the corresponding author upon request.
