# Supplementary material for: Responses of the tree peony (Paeonia suffruticosa, Paeoniaceae) cultivar ‘Yu Hong’ to heat stress revealed by iTRAQ-based quantitative proteomics
Source: Proteome Sci. 2022 Dec 29;20:18. doi: 10.1186/s12953-022-00202-5 (PMC9798725; doi:10.1186/s12953-022-00202-5)
Supplement: Supplementary file 1 — Additional file 1: Schedule 1. Significantly expressed differential proteins under high-temperaturestress. [file 12953_2022_202_MOESM1_ESM.docx]

Schedule 1 Significantly expressed differential proteins under high-temperature stress

| **Protein ID** | **Protein names** | **Fold change** |
| --- | --- | --- |
| Protein synthesis, processing, and degradation | | |
| c104797.graph_c0 | E2-17Ka Ubiquitin-binding enzyme E2-17Ka | 2.593 |
| c93677.graph_c0 | Ubiquitin-like family protein | 2.276 |
| c103417.graph_c0 | Chaperone-protein ClpB | 2.245 |
| c115415.graph_c1 | Peptidylprolyl isomerase | 2.23 |
| c118249.graph_c1 | Chaperone-protein ClpB | 1.858 |
| c111758.graph_c0 | Peptidylprolyl isomerase | 1.686 |
| c110207.graph_c0 | 60S ribosomal protein L26-1 | 1.677 |
| c116290.graph_c3 | 40S Ribosomal protein | 1.605 |
| c106105.graph_c0 | Peptidylprolyl isomerase | 1.578 |
| c117236.graph_c1 | Aspartate protease family protein | 0.665 |
| c108032.graph_c0 | Trigger factor protein | 0.664 |
| c117276.graph_c1 | Peptidine family protein | 0.515 |
| c92427.graph_c0 | Subtilisin-like serine protease | 0.491 |
| c112740.graph_c0 | Protein disulfide isomerase | 0.335 |
| Heat shock protein | | |
| c107551.graph_c0 | Small heat shook protein | 8.445 |
| c117526.graph_c2 | Small heat shook protein | 4.88 |
| c120033.graph_c1 | Small heat shook protein | 4.261 |
| c112621.graph_c0 | Small heat shook protein | 4.133 |
| c117374.graph_c1 | Small heat shook protein | 3.918 |
| c112949.graph_c1 | Small heat shook protein | 3.643 |
| c104422.graph_c0 | Small heat shook protein | 3.299 |
| c100675.graph_c0 | Small heat shook protein | 3.249 |
| c116391.graph_c0 | Small heat shook protein | 2.924 |
| c92835.graph_c0 | Small heat shook protein | 2.723 |
| c110265.graph_c0 | Small heat shook protein | 2.693 |
| c94118.graph_c0 | Small heat shook protein | 2.692 |
| c114184.graph_c1 | Small heat shook protein | 2.494 |
| c114184.graph_c0 | Small heat shook protein | 2.071 |
| c119976.graph_c0 | Hsp70 family protein | 2.652 |
| c65824.graph_c0 | Hsp70 family protein | 2.479 |
| c110893.graph_c1 | Hsp70 family protein | 2.343 |
| c17729.graph_c0 | Hsp70 family protein | 2.082 |
| c112482.graph_c0 | Hsp70 family protein | 1.804 |
| c114151.graph_c1 | Hsp70 family protein | 1.508 |
| c101826.graph_c1 | Hsp90 family protein | 6.893 |
| c119657.graph_c2 | Hsp90 family protein | 3.594 |
| c120096.graph_c0 | Hsp90 family protein | 1.568 |
| Defense and antioxidants | | |
| c100066.graph_c1 | Peroxidase | 4.54 |
| c115075.graph_c0 | Ascorbate peroxidase | 2.945 |
| c114484.graph_c0 | Glutathione peroxidase | 2.65 |
| c116374.graph_c0 | Catalase | 2.123 |
| c118463.graph_c0 | 2-alkenal reductase | 2.042 |
| c119317.graph_c2 | Pathogen-related proteins | 0.64 |
| c119749.graph_c0 | Pathogenesis-related proteins STH-21 | 0.571 |
| c102656.graph_c0 | NADPH-protochlorophyllide oxidoreductase | 0.405 |
| Energy production and conversion | | |
| c104351.graph_c0 | Citrate synthase | 3.65 |
| c41383.graph_c0 | Fructose- 1,6- bisphosphate aldolase | 3.552 |
| c117676.graph_c3 | Phosphoglycerate kinase | 1.879 |
| c112840.graph_c0 | Malate dehydrogenase | 1.813 |
| c119073.graph_c0 | Cytochrome C oxidase | 1.805 |
| c112084.graph_c0 | Inorganic pyrophosphatase | 0.654 |
| c115173.graph_c0 | Dihydrolipoyl dehydrogenase | 0.649 |
| c102938.graph_c1 | Adenylate kinase | 0.421 |
| c106302.graph_c0 | NAD Oxidoreductase | 0.243 |
| Signal transduction | | |
| c116136.graph_c0 | Highlight fusion protein | 4.405 |
| c113074.graph_c1 | ABC transporter family member | 3.391 |
| c109749.graph_c1 | Calmodulin | 1.764 |
| c119803.graph_c0 | Calcium-dependent protein kinase | 1.605 |
| c103057.graph_c0 | Beta family protein | 0.635 |
| c110394.graph_c0 | Protein kinase containing aarF domain | 0.608 |
| c108336.graph_c0 | STNT Serine/threonine protein kinase STNT | 0.42 |
| c117798.graph_c0 | Purple acid phosphatase | 0.528 |
| Photosynthesis | | |
| c96149.graph_c0 | Chlorophyll a / b binding protein | 3.587 |
| c116330.graph_c0 | NADH dehydrogenase | 3.07 |
| c108824.graph_c0 | FtsH protease | 1.791 |
| c118450.graph_c0 | P450 Cytochrome P450 | 1.641 |
| c113011.graph_c0 | Oxygenation enhancing protein | 0.648 |
| c110422.graph_c1 | Ribonose-1,5- diphosphate-carboxylase/oxygenase | 0.613 |
| c106604.graph_c0 | Ribonose-1,5- diphosphate- carboxylase/oxygenase | 0.517 |
| Metabolism | | |
| c111030.graph_c0 | Glutamine Synthetase | 1.523 |
| c117414.graph_c0 | Phenylalanine ammonia lyase | 2.749 |
| c117871.graph_c2 | Acetyl-CoA carboxylase | 2.54 |
| c125409.graph_c0 | Alcohol dehydrogenase | 0.641 |
| c116660.graph_c0 | Serine carboxypeptidase | 0.654 |
| c114809.graph_c3 | Cell lytic oligopeptidas | 0.652 |
| c110244.graph_c0 | Arginase | 0.634 |
| c109559.graph_c0 | Adenylyl sulfate reductase 3 | 0.603 |
| c118812.graph_c0 | UDP-glucosyltransferase | 0.59 |
| c109213.graph_c0 | Keto alcohol reductoisomerase | 0.572 |
| c113833.graph_c0 | Serine carboxypeptidase | 0.566 |
| c110119.graph_c0 | Serine carboxypeptidase | 0.496 |
| c111391.graph_c0 | Diaminopimelate decarboxylase | 0.468 |
| Other differential proteins | | |
| c117737.graph_c0 | Retrotran sposon gag protein | 5.882 |
| c119494.graph_c1 | Splicing factor 3B subunit | 1.996 |
| c105385.graph_c0 | 4-Hydroxy 3-methylbutyl 2-enyl diphosphate reductase | 0.654 |
| c103603.graph_c0 | NBRI protein homolog | 0.608 |
| c97902.graph_c0 | D-C-methyl-D-erythritol 4-phosphatidyltransferase | 0.605 |
| c80494.graph_c0 | Microtubulin α-2 chain | 0.582 |
| c100473.graph_c0 | Enoyl-acyl carrier protein | 0.561 |
| c100811.graph_c0 | Aquaporin TIP1-1 | 0.53 |
| c56017.graph_c0 | Bridging integration factor protein | 0.528 |
| c116808.graph_c0 | PsbP domain contains protein I | 0.523 |
| c111633.graph_c0 | Unidentified protein | 0.491 |
| c106360.graph_c0 | TL20.3 Thylakoid lumenal protein TL20.3 | 0.479 |
| c100168.graph_c0 | Dihydropyrimidine dehydrogenase | 0.461 |
| c112298.graph_c0 | NAD-dependent epimerase | 0.375 |
| c100532.graph_c0 | Lysis transglycosylase | 0.341 |
| c106213.graph_c0 | Vacuolar-processing enzyme | 0.246 |
| c111744.graph_c0 | CBOM-04674 Hypothetical protein CBOM-04674 | 0.665 |
| c96097.graph_c0 | Basic 7S globulin | 0.635 |
